# Supplementary material for: A network dynamical simulation model for the study of antibiotic resistance in microbial communities
Source: Gut Microbes. 2026 Sep 19;18(1):2734703. doi: 10.1080/19490976.2026.2734703 (PMC13596919; doi:10.1080/19490976.2026.2734703)
Supplement: Supplementary file 1 [file KGMI_A_2734703_SM9053.zip › supplementary_file_1/Figures/real_pct_delta_n_edges.pdf]

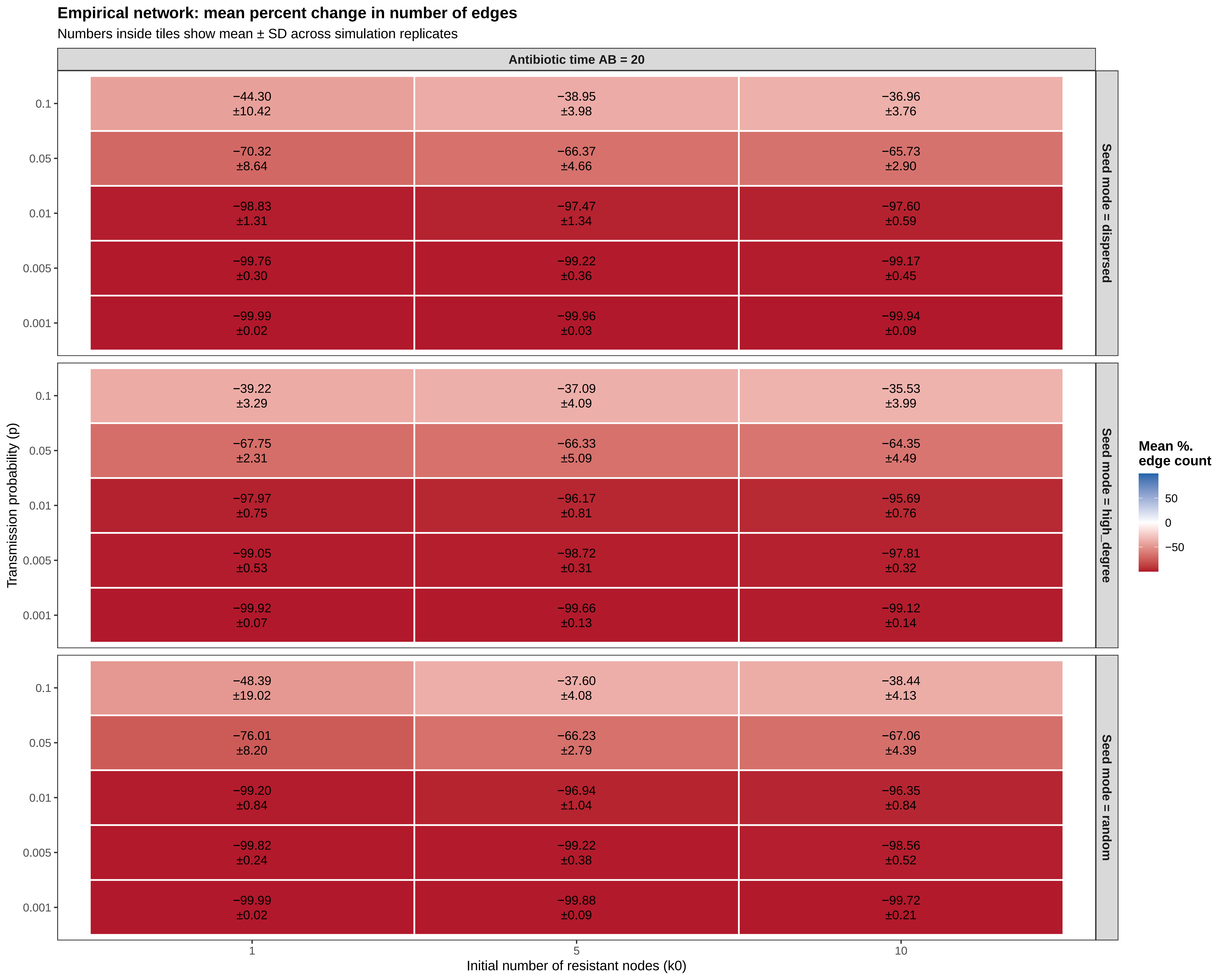
Empirical network: mean percent change in number of edges
Numbers inside tiles show mean ± SD across simulation replicates
Antibiotic time AB = 20
Seed mode = dispersed
0.1
−44.30 ±10.42
−38.95 ±3.98
−36.96 ±3.76
0.05
−70.32 ±8.64
−66.37 ±4.66
−65.73 ±2.90
0.01
−98.83 ±1.31
−97.47 ±1.34
−97.60 ±0.59
0.005
−99.76 ±0.30
−99.22 ±0.36
−99.17 ±0.45
0.001
−99.99 ±0.02
−99.96 ±0.03
−99.94 ±0.09
Seed mode = high_degree
0.1
−39.22 ±3.29
−37.09 ±4.09
−35.53 ±3.99
0.05
−67.75 ±2.31
−66.33 ±5.09
−64.35 ±4.49
0.01
−97.97 ±0.75
−96.17 ±0.81
−95.69 ±0.76
0.005
−99.05 ±0.53
−98.72 ±0.31
−97.81 ±0.32
0.001
−99.92 ±0.07
−99.66 ±0.13
−99.12 ±0.14
Seed mode = random
0.1
−48.39 ±19.02
−37.60 ±4.08
−38.44 ±4.13
0.05
−76.01 ±8.20
−66.23 ±2.79
−67.06 ±4.39
0.01
−99.20 ±0.84
−96.94 ±1.04
−96.35 ±0.84
0.005
−99.82 ±0.24
−99.22 ±0.38
−98.56 ±0.52
0.001
−99.99 ±0.02
−99.88 ±0.09
−99.72 ±0.21
Transmission probability (p)
1
5
10
Initial number of resistant nodes (k0)
Mean %.
edge count
50
0
−50
